# Synthesis and anticancer activity of Pt(iv) prodrugs containing 3-bromopyruvic acid as an axial ligand†

**DOI:** 10.1039/d5ra02064f

**Published:** 2025-06-30

**Authors:** Peng Zhou, Qingfeng Xiong, Ling Zhang, Jing Jiang, Yan Song, Weiping Liu, Limei Zhang, Anli Gao, Chen Qing

**Affiliations:** a School of Pharmaceutical Science and Yunnan Key Laboratory of Pharmacology for Natural Products, Kunming Medical University Kunming City 650500 China qingchenhhh@qq.com; b Department of Pharmacy, The Second Affiliated Hospital of Kunming Medical University 374 Dianmian Avenue Kunming City 650101 China; c State Key Laboratory of Precious Metal Functional Materials 988 Keji Road Kunming City 650106 China gaoanli@126.com

## Abstract

A Pt(iv) prodrug of oxaliplatin incorporating the glycolysis inhibitor 3-bromopyruvic acid, BrPt3, was designed and investigated. The prodrug is reduced in the presence of ascorbic acid, releasing its active Pt(ii) species and axial ligand. In cytotoxicity studies, BrPt3 exhibited stronger anticancer activity than oxaliplatin against all tested cancer cell lines, particularly in oxaliplatin-resistant A549/OXP cells. By effectively inhibiting glycolysis, BrPt3 induced greater DNA damage in tumor cells. It arrested the cell cycle at the G0/G1 phase, leading to increased apoptosis and significantly reduced cell invasiveness. BrPt3 resulted in higher platinum accumulation in the genomic DNA of MKN28, HCT116, and HT29 cells, though no significant difference in DNA-platinum adduct formation was observed in A549/OXP cells. Compared to oxaliplatin, BrPt3 more effectively impaired the glycolytic capacity of tumor cells, as evidenced by significantly reduced levels of pyruvate, lactate, ATP, and HK2 enzyme expression. *In vivo* studies using an HCT116 xenograft tumor mouse model demonstrated that BrPt3 achieved a higher tumor inhibition rate and lower toxicity than oxaliplatin. Although gavage administration resulted in a lower tumor inhibition rate than intraperitoneal administration, it still exhibited substantial antitumor activity. Overall, this Pt(iv) prodrug holds potential for development as an oral anticancer agent through its dual mechanisms of inducing DNA damage and inhibiting glycolysis.

## Abstract 2

The 3-bromopyruvic acid platinum(iv) complex BrPt3 demonstrates significant antitumor efficacy through dual mechanisms: inducing DNA damage and inhibiting glycolysis.

## Graphical abstract

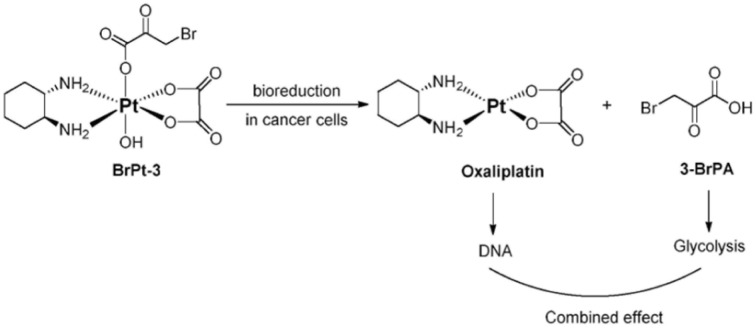

## Introduction

In 1965, Rosenberg accidently discovered that cisplatin, formed through platinum electrode electrolysis, inhibited cell division while studying the effects of electric fields on bacteria. This finding led to the development of platinum-based anticancer drugs.^1^ Cisplatin, the first of its kind, was approved by the FDA in 1978 for the treatment of testicular, ovarian, and bladder cancers, marking a milestone in cancer therapy. Carboplatin, with lower toxicity, is primarily used for ovarian cancer, while oxaliplatin (OXP) is effective against metastatic colorectal cancer.^2,3^ Cisplatin, carboplatin, and OXP are Pt(ii) compounds that induce apoptosis by binding to purine bases in DNA, disrupting replication and transcription, thereby effectively targeting rapidly dividing cancer cells.^4,5^ Despite their clinical success, these drugs have limitations, including nephrotoxicity, myelosuppression, and drug resistance, which arise from enhanced DNA repair mechanisms and altered drug uptake or efflux.^6,7^ The development of new platinum-based drugs aims to improve efficacy and reduce adverse effects to address these challenges.

Pt(iv) compounds have gained attention in cancer therapy due to their unique properties. Their d_6_ electronic configuration and octahedral geometry provide higher stability, leading to slower reduction and a prolonged half-life *in vivo*.^8^ This stability reduces rapid degradation and toxicity, as the active Pt(ii) species is only released within cancer cells. The reduction to Pt(ii), facilitated by intracellular agents such as glutathione and ascorbic acid, enables effective DNA binding and anticancer activity, with the reaction rate adjustable through ligand modifications.^9^ Pt(iv) compounds are notable for their multifunctionality, allowing the introduction of additional biological activities by incorporating various ligands at their axial positions.^10^ This feature enables them to function through multiple mechanisms beyond DNA damage. Their structural flexibility offers promising potential in cancer therapy, allowing the design of Pt(iv) compounds with enhanced selectivity, efficacy, and the ability to overcome platinum resistance.^11–13^

In normal cells, energy is primarily derived from mitochondrial aerobic metabolism, shifting to glycolysis under hypoxic conditions and returning to aerobic metabolism when oxygen becomes available. However, tumor cells rely on glycolysis for energy production regardless of oxygen availability, a phenomenon known as the Warburg effect.^14–18^ Glycolysis has become a key target in cancer research.^19,20^ 3-Bromopyruvic acid (BP) inhibits hexokinase II, disrupting tumor cell glycolysis and inducing apoptosis, making it a promising anticancer agent.^21^ Studies have shown that BP can counteract multidrug resistance in tumors and enhance the efficacy of various anticancer drugs, including platinum-based agents.^22,23^ However, the stability of BP remains a major obstacle to its therapeutic application *in vivo*.^24,25^ To improve the dual-targeting effects of OXP and BP, 3-bromopyruvic acid was incorporated to inhibit both DNA replication and glycolysis in tumor cells while modifying OXP's structure to enhance oral bioavailability.

## Results and discussion

### Chemistry

**Fig. 1:**
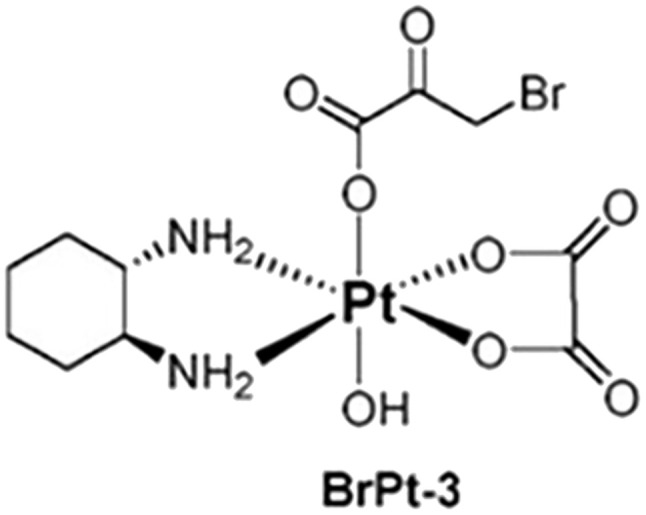
Chemical structure of BrPt3.

BrPt3 was synthesized using OXP as the starting material. Initially, OXP underwent axial oxidation with 30 wt% hydrogen peroxide, followed by a reaction with an excess of BP in an aqueous solution. The reaction mixture was then concentrated under reduced pressure to near dryness. The solid residue was filtered and subjected to sequential washing with ice-cold acetone and ice-cold ethanol. The final product, BrPt3, was obtained by drying under vacuum conditions. The structure of BrPt3 (Fig. 1) was characterized using elemental analysis, ^1^H and ^13^C nuclear magnetic resonance (NMR) spectroscopy, infrared (IR) spectroscopy and fast atom bombardment mass spectrometry (FAB^+^-MS). These values aligned precisely with the anticipated molecular formulas, as corroborated by the data presented in Fig. S3 to S6.†

### Reduction behavior

**Fig. 2:**
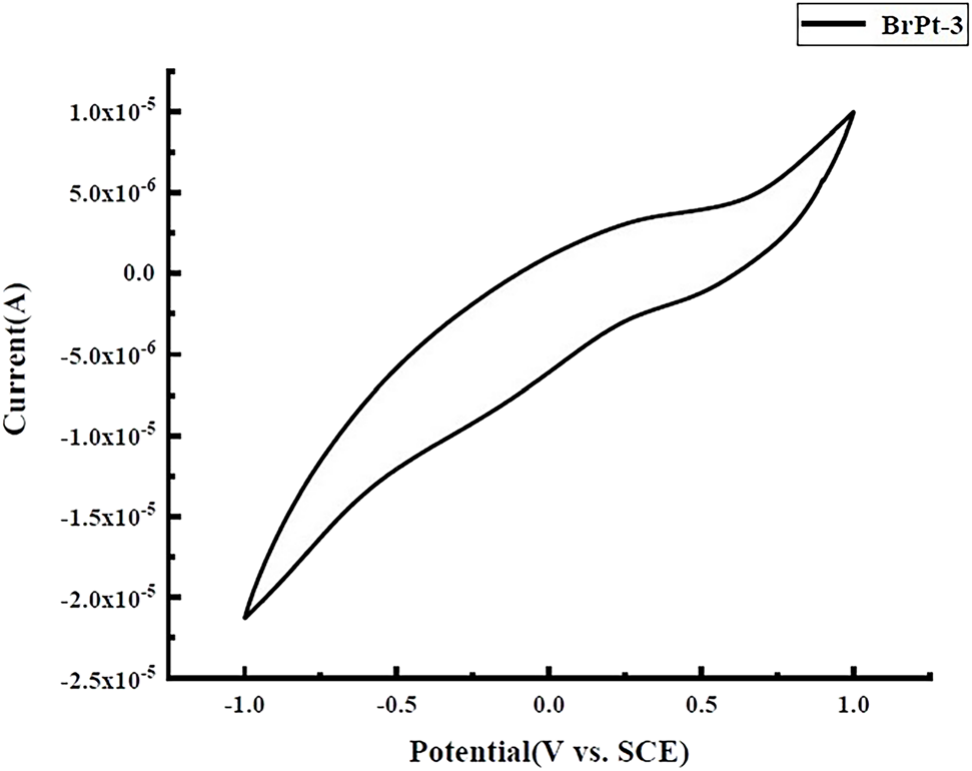
Cyclic voltammograms of BrPt3.

Unlike Pt(ii) compounds, Pt(iv) prodrugs do not directly bind to DNA or nucleophiles, making their redox potential a key factor in determining their antitumor activity. High-performance liquid chromatography (HPLC) and cyclic voltammetry analyses confirmed that the 3-bromopyruvic acid Pt(iv) prodrug is reduced to its active Pt(ii) form, which exerts antitumor effects.^26^Fig. 2 shows the cyclic voltammograms of BrPt3 in an aqueous solution of 0.1 mM NaCl, revealing an irreversible reduction with a peak potential (*E*_p_) of −0.17 V. This indicates that BrPt3 readily reduces to its active Pt(ii) form, which is essential for its antitumor effects. The waves in the CVs in Fig. 2 are not sharp, the possible factors include the electrode surface state, electrolyte composition, and the kinetics of the electrochemical reactions. The presence of impurities or an uneven distribution of active sites on the electrode surface may hinder electron transfer. The concentration and ionic strength of the electrolyte solution, as well as any impurities present, could also interfere with the electrochemical reactions, altering their kinetics and thus the shape of the CV curves.

**Fig. 3:**
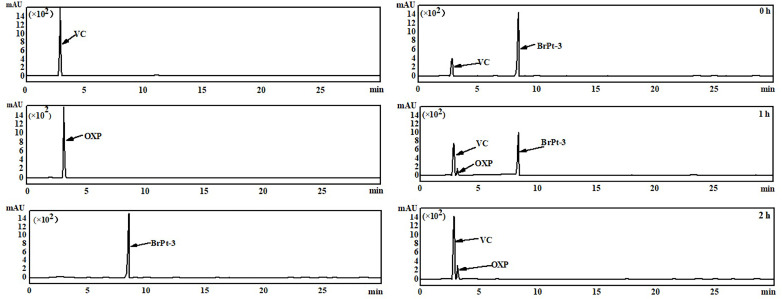
HPLC chromatogram of BrPt3 treated with vitamin C.

As shown in Fig. 3, the left panel displays the HPLC chromatograms of vitamin C, OXP, and BrPt3, respectively. The right panel illustrates the chromatograms of OXP and vitamin C incubated at 37 °C for 0, 1, and 2 hours. At 0 h, only the characteristic peaks of vitamin C and BrPt3 were observed. After 1 h, the BrPt3 peak decreased significantly, accompanied by a marked increase in the OXP peak. By 2 h, the BrPt3 peak had completely disappeared, while the OXP peak further increased compared to the 1 h time point. This suggests that BrPt3, once absorbed by cells, undergoes rapid reduction by intracellular reducing agents such as vitamin C, leading to the release of the Pt(ii) complex, which exerts its antitumor effects.

### Cytotoxicity

BrPt3 effectively inhibited tumor cell growth and demonstrated higher inhibitory activity against A549/OXP cells compared to the original drug. However, its cytotoxic effect on L02 cells was consistently lower than that of the original drug, indicating selectivity. The incorporation of BP as an axial ligand enhances the efficacy, cytotoxicity, and selectivity of the prodrug while also reversing OXP resistance (Table 1).

**Table 1:** *In vitro* cytotoxicity (IC_50_ µM) of BrPt3 against human cancer cell lines

Cell line	OXP	BrPt3
A549	18.22 ± 0.80	11.72 ± 1.49
MKN28	76.41 ± 12.72	47.28 ± 5.90
HCT116	27.09 ± 1.40	14.03 ± 0.80
HepG2	29.66 ± 1.32	11.69 ± 2.63
HT29	29.70 ± 0.35	16.67 ± 0.29
A549/OXP	211.61 ± 22.18	66.76 ± 6.07
L02	9.78 ± 0.60	13.37 ± 0.12

### *In vitro* anticancer activity

**Fig. 4:**
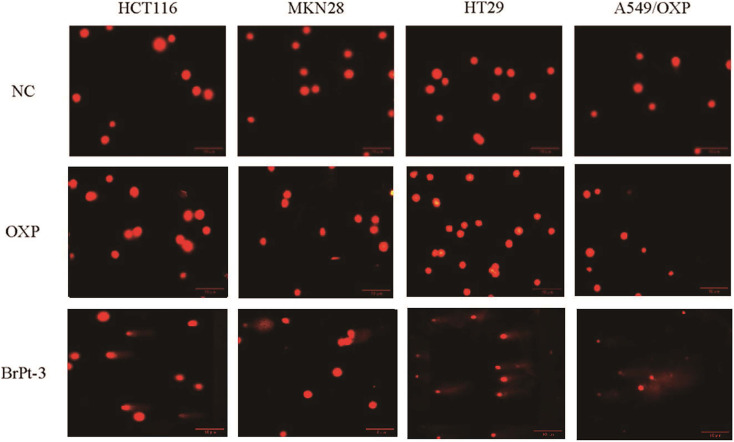
Representative images of nuclei in the comet assay of OXP and BrPt3 in HCT116, MKN28, HT29, and A549/OXP cells. Scale bars = 10 µm.

**Fig. 5:**
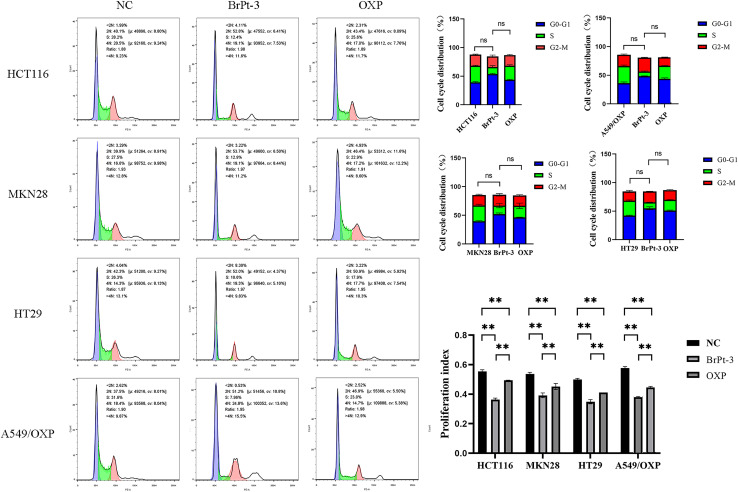
Cell cycle arrest analysis of OXP and BrPt3 in HCT116, MKN28, HT29, and A549/OXP cells. Data are presented as mean ± SD from three independent experiments, each performed in triplicate. **P < 0.01.

**Fig. 6:**
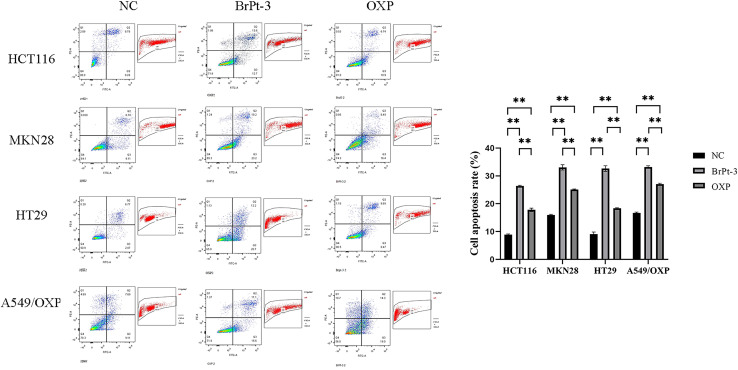
*In vitro* apoptosis of cancer cells pretreated with OXP and BrPt3. Data are presented as mean ± SD from three independent experiments, each performed in triplicate. **P < 0.01.

**Fig. 7:**
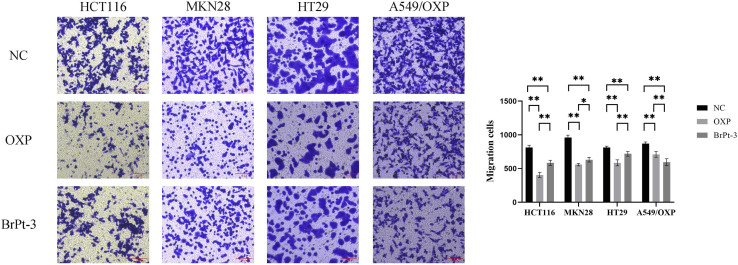
Transwell assay for evaluating cell invasion pretreated with OXP and BrPt3. Scale bars = 10 µm. Data are presented as mean ± SD from three independent experiments, each performed in triplicate. *P < 0.05, **P < 0.01.

Platinum-based drugs primarily target DNA, and their therapeutic effectiveness depends on inducing genomic damage in tumor cells. Therefore, evaluating drug-induced DNA damage is essential for understanding their mechanism of action. Single-cell gel electrophoresis enables the visual assessment of DNA damage by analyzing DNA migration under an electric field.^27^ Tumor cells treated with BrPt3 exhibited longer DNA tails, indicating significant DNA strand breaks. Fig. 4 shows that BrPt3 significantly induces DNA damage and strand breaks in tumor cells, as evidenced by the presence of a trailing pattern. As shown in Fig. 5, BrPt3 primarily induced G0/G1 phase cell cycle arrest, preventing progression to the S phase and extending the duration of the G1 phase, thereby preventing cell proliferation and inhibiting tumor growth. Similar effects have been observed with resveratrol and docetaxel, which activate p53 and upregulate p21 and p27, leading to G0/G1 phase blockade.^28,29^ Unlike OXP, Fig. 6 shows that BrPt3 induced significantly higher apoptosis rates in all four tumor cell lines tested at the same concentration over a 48-hour period. Tumor development is often associated with the suppression of apoptosis. Many clinical anticancer drugs function by inducing apoptosis, making the study of apoptotic pathways crucial for cancer therapy.^30,31^Fig. 7 shows that BrPt3 significantly reduces the invasive capacity of HCT116, MKN28, HT29, and A549/OXP cells after 48 hours of treatment. Tumor invasiveness is a major factor contributing to metastasis and recurrence, which are associated with poor clinical outcomes.^32^ Transwell invasion assays demonstrated that while OXP promoted tumor cell penetration, BrPt3 significantly reduced it, indicating a stronger ability to inhibit tumor cell migration and invasion.

### DNA platination

Platinum-based drugs enter cells through active transport or passive diffusion, and their uptake efficiency influences tumor cell sensitivity to treatment. To clearly demonstrate the transport mechanism of platinum complexes, platinum content in genomic DNA was measured using ICP-MS.^33–35^ The effect of BrPt3 on cellular uptake was assessed by measuring platinum content in genomic DNA across various cell lines. Table 2 shows that in MKN28 cells, BrPt3 resulted in a platinum content 14.63 times higher than that of an equimolar concentration of OXP. In HCT116 and HT29 cells, platinum content was 2.05 and 1.72 times higher, respectively, indicating a correlation between BrPt3-induced cytotoxicity and DNA-Pt binding. In contrast, A549/OXP cells showed no significant difference in DNA-Pt binding when treated with BrPt3. In IOSE cells, platinum content with BrPt3 was significantly lower, reaching only 3.50% of that observed with an equimolar concentration of OXP, suggesting a reduced impact on normal cells compared to OXP. BrPt3 exhibited high platinum accumulation in the DNA of MKN28, HCT116, and HT29 cells, correlating its strong cytotoxicity with efficient cellular uptake and DNA binding. In contrast, BrPt3 showed lower DNA-Pt binding in normal IOSE cells than OXP, suggesting higher selectivity. However, in A549/OXP cells, DNA-Pt binding levels were similar between BrPt3 and OXP, indicating that its enhanced cytotoxicity in drug-resistant cells may be attributed to the BP ligand's role in overcoming drug resistance.

**Table 2:** Comparison of platinum content in the genomic DNA of different cells for OXP and BrPt3 (*n* = 3)

Drug	Pt (ng per mg DNA)				
	MKN28	HCT116	HT29	A549/OXP	IOSE
OXP	0.16 ± 0.09	0.22 ± 0.16	0.89 ± 0.12	0.05 ± 0.01	6.58 ± 0.46
BrPt3	2.34 ± 1.20	0.45 ± 0.20	1.53 ± 0.20	0.06 ± 0.05	0.23 ± 0.01

### Glycolytic capacity

**Fig. 8:**
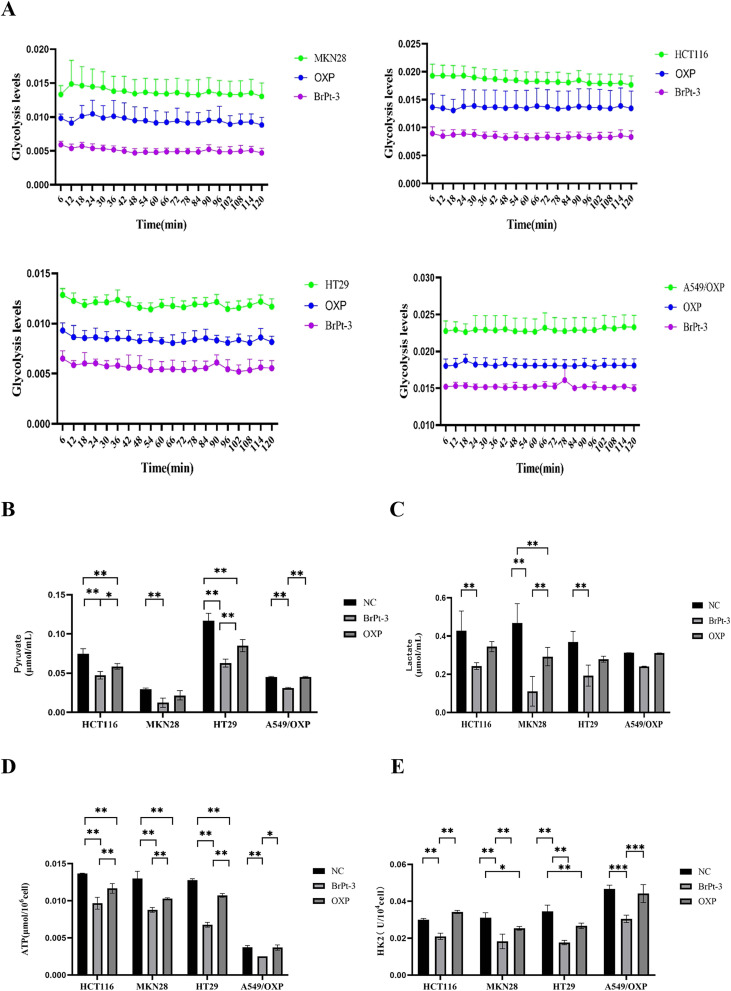
Effects of OXP and BrPt3 on glycolysis. (A) Extracellular acidification rate (ECAR) curve used to calculate ECAR. (B) Pyruvate levels. (C) Lactate levels. (D) ATP levels. (E) HK2 levels. Data are presented as mean ± SD from three independent experiments, each performed in triplicate. *P < 0.05, **P < 0.01, ***P < 0.001.

Tumor cells primarily depend on glycolysis for energy production, generating ATP and essential metabolic intermediates. Abnormal glucose metabolism in cancer cells is closely linked to metastasis, tumor growth, and drug resistance. Specific glycolysis-related enzymes contribute to drug resistance by regulating glycolytic activity.^36,37^ HK2, a key enzyme in glycolysis, catalyzes the conversion of glucose to glucose-6-phosphate, playing a central role in tumor metabolism and the Warburg effect, making it an important therapeutic target.^38^ To evaluate changes in energy metabolism during BrPt3-induced tumor cell death, intracellular glycolysis levels were measured using a glycolysis assay kit. Fig. 8 shows that OXP significantly reduced glycolysis in tumor cells, leading to decreased levels of pyruvate, lactate, ATP, and HK2. BrPt3 further inhibited cellular glycolysis compared to OXP.

### *In vivo* antitumor effects

**Fig. 9:**
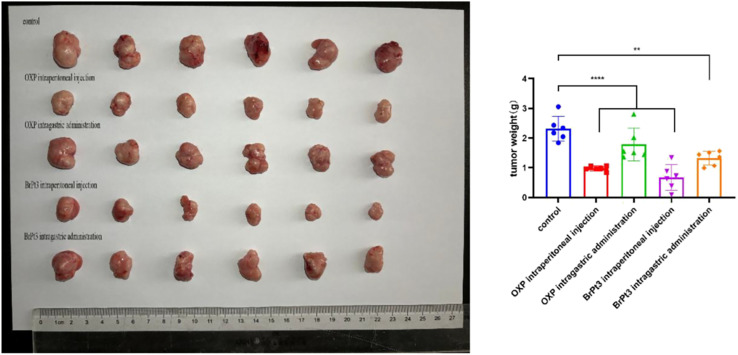
Representative images of tumors from each group at the end of the experiment and statistical analysis of tumor weights. Statistical significance was determined using a two-way ANOVA test (**P < 0.01, ****P < 0.0001).

**Fig. 10:**
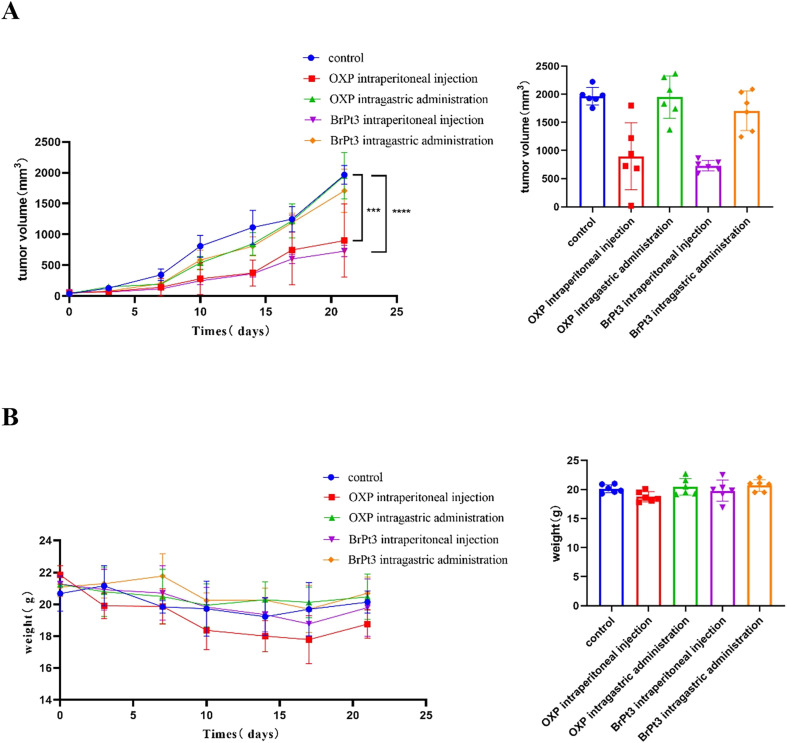
*In vivo* antitumor activity of BrPt3 in HCT116 xenograft tumors. (A) Tumor growth in mice and statistical analysis of tumor volume over the experimental period. (B) Statistical analysis of body weight fluctuations in mice during the experiment. Statistical significance was determined using a two-way ANOVA test (***P < 0.001, ****P < 0.0001).

**Fig. 11:**
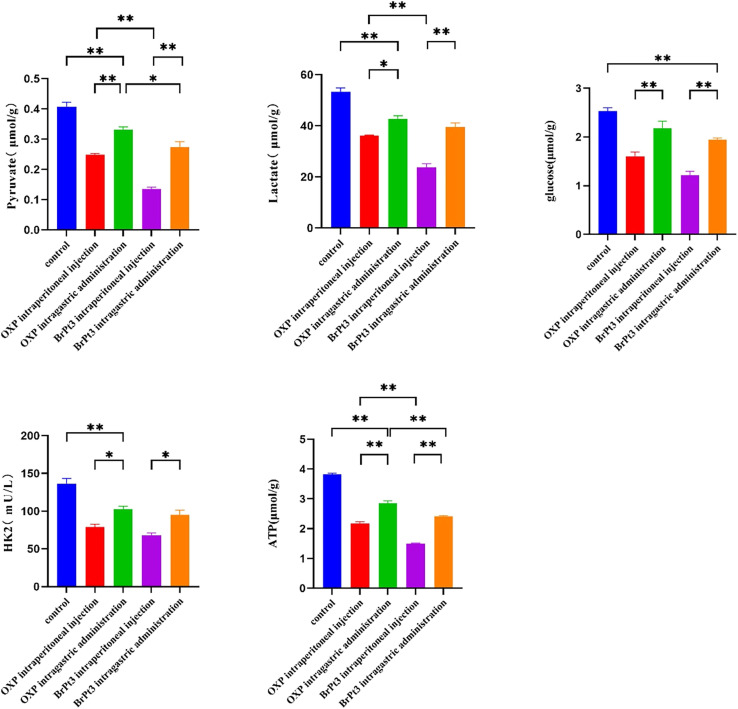
Glucose, lactate, pyruvate, ATP, and HK2 levels in tumor tissue homogenates. Statistical significance was determined using a two-way ANOVA test (*P < 0.05, **P < 0.01).

Fig. 9 and Table S1† shows that BrPt3 exhibited stronger tumor growth inhibition in HCT116 xenograft nude mice compared to OXP, with inhibition rates of 70.56% for intraperitoneal administration and 42.78% for gavage administration, exceeding the inhibition rates of 57.58% and 22.79% for OXP, respectively. These findings align with *in vitro* results. According to Fig. 10A and Table S2,† OXP treatment in HCT116 tumor-bearing nude mice resulted in tumor proliferation rates of 33.46% for intraperitoneal administration and 82.70% for gavage administration, showing significant tumor growth inhibition only in the intraperitoneal group. In contrast, BrPt3 treatment resulted in tumor proliferation rates of 21.67% intraperitoneally and 55.08% by gavage, demonstrating stronger tumor suppression compared to OXP. As shown in Fig. 10B, mice in the NC group exhibited weight gain and tumor growth. In the OXP intraperitoneal administration group, nude mice experienced significant weight loss compared to the start of the experiment, whereas weight changes in other groups were minimal compared to the NC group. Fig. 11 demonstrates that BrPt3 significantly reduced glucose, lactate, pyruvate, ATP, and HK2 levels, suggesting its potential antitumor effects *via* glycolysis inhibition.

**Fig. 12:**
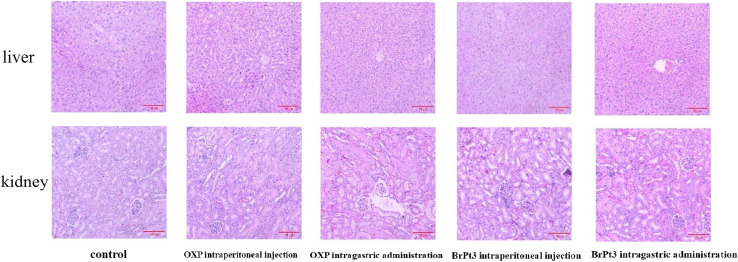
Representative images of H&E-stained liver and kidney tissues. Scale bars = 10 µm.

**Fig. 13:**
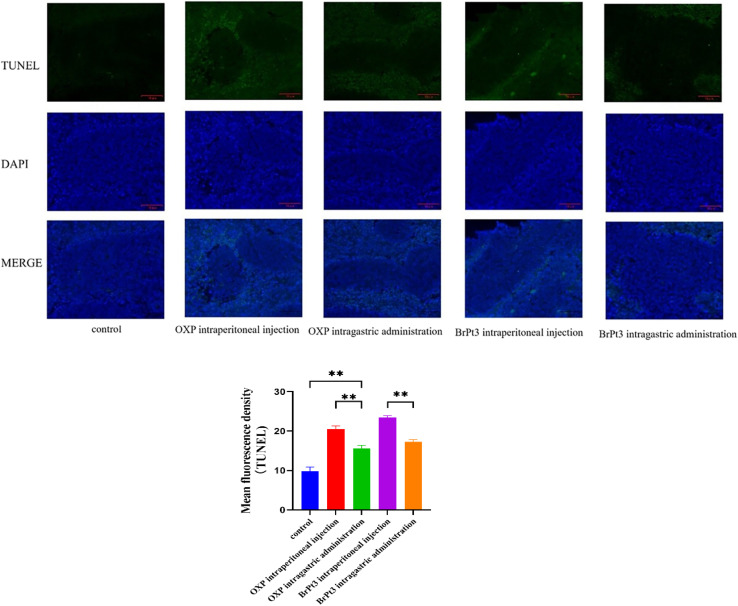
Representative images of TUNEL staining in tumor tissues. Scale bars = 10 µm. Statistical significance was determined using a two-way ANOVA test (**P < 0.01).

**Fig. 14:**
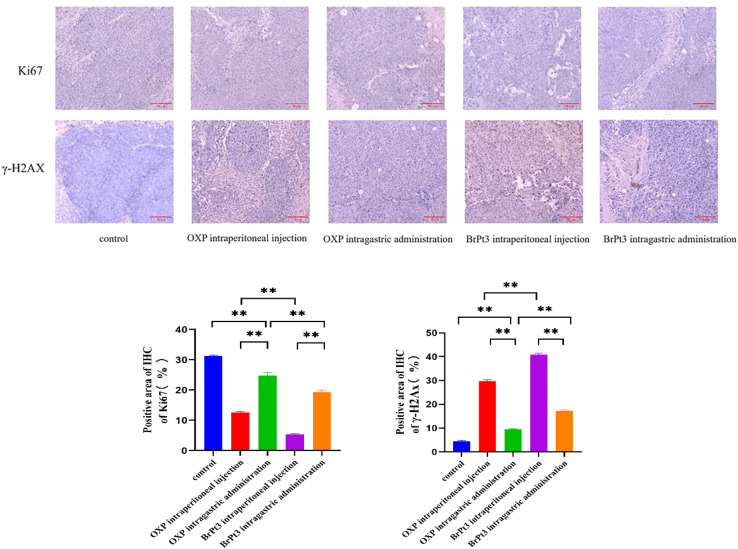
Representative images of Ki67 and γ-H2AX staining in tumor tissues. Scale bars = 10 µm. Statistical significance was determined using a two-way ANOVA test (**P < 0.01).

For BrPt3 safety evaluation, hematoxylin-eosin (HE) staining of liver and kidney tissues in nude mice was performed. Liver histology in the NC group appeared normal, with well-defined central veins and structured hepatocytes. In the OXP-treated group, although the hepatic architecture remained intact, some hepatocytes were enlarged and swollen, with lighter cytoplasmic staining and observed lymphocytic infiltration. Fig. 12 shows that kidney histology in the NC group was normal, displaying a well-organized renal tubule structure. In contrast, the OXP intraperitoneal and gavage groups exhibited irregular renal tubule morphology, swollen epithelial cells, reduced cytoplasm, and fewer nuclei. These effects were more pronounced in the gavage group, which also displayed numerous tubular patterns. The BrPt3 intraperitoneal group showed milder changes, with clearer and more regular renal tubule structures and only slight cell swelling. The BrPt3 gavage group exhibited even fewer alterations, closely resembling the NC group's histomorphology. Fig. 13 demonstrates that TUNEL staining revealed a higher number of apoptotic cells in BrPt3-treated tumors than in OXP-treated tumors. Immunohistochemistry was performed to assess Ki67 and γ-H2AX protein levels in transplanted tumors. Fig. 14 indicates that Ki67 expression decreased, while γ-H2AX levels significantly increased in both the BrPt3 intraperitoneal and gavage groups compared to the OXP group. BrPt3 significantly inhibited tumor cell proliferation while increasing apoptosis and DNA damage, as demonstrated by γ-H2AX and Ki67 immunohistochemistry and TUNEL staining.

## Experimental

### Chemical synthesis

See ESI† for synthetic methods.

### High performance liquid chromatography

Dissolve the Pt(iv) precursor in a phosphate-buffered saline (PBS) and acetonitrile solution (pH 7.4, 40 : 60 V/V). Prepare a 50.0 µM vitamin C solution in deionized water using ultrasonic dissolution and filtration. Mix equal volumes of the Pt(iv) precursor and vitamin C solutions, then incubate the mixture in a 37 °C water bath, protected from light. Collect reaction samples at 0, 1, and 2 hours for high-performance liquid chromatography (HPLC) analysis under the specified mobile phase conditions.

### Cyclic voltammetry

The electrochemical behavior of Pt(iv) complexes was analyzed using a standard three-electrode system, consisting of a 3 mm glassy carbon working electrode, a platinum wire counter electrode, and a saturated calomel reference electrode. The aqueous test solution contained 0.1 mM target compound and 0.1 mM NaCl. Cyclic voltammetry was performed at 25 °C with a scan rate of 100 mV s^−1^ using a BAS100 workstation. The potential window ranged from −1.0 V to +1.0 V (*vs.* SCE), starting at +0.9 V. Before testing, the working electrodes were polished with alumina, and nitrogen gas was used to remove dissolved oxygen for 15 minutes. Data were collected at 1 mV intervals, with each sample measured three times to ensure reproducibility.

### Cell culture

Human cancer cell lines A549, MCF7, HCT116, MKN28, HepG2, HT29, and LO2 were cultured in DMEM or RPMI-1640 medium, supplemented with 10% fetal bovine serum, 100 units per mL penicillin, and 100 µg per mL streptomycin. The cells were maintained at 37 °C in a 5% CO_2_ incubator.

### Cytotoxic activity

Cells in the logarithmic growth phase were digested, prepared into single-cell suspensions, counted, and adjusted to a specific concentration. The suspensions were inoculated into 96-well plates at 90 µL per well and incubated for 24 hours to allow cell adhesion. After adhesion, drugs were added, and incubation continued for another 48 hours. Subsequently, 20 µL of 5 mg per mL MTT solution was added to each well, followed by a 4-hour incubation. After incubation, the supernatant was removed, and 100 µL of DMSO was added to dissolve the formazan product. Absorbance was measured at 570 nm with a reference wavelength of 630 nm using a microplate reader. Each sample group was tested in triplicate.

### Single-cell gel electrophoresis

See ESI† for single-cell gel electrophoresis.

### Cell apoptosis and cell cycle arrest

For cell apoptosis analysis, cells were treated with trypsin, resuspended in Annexin V binding buffer, stained with Annexin V-FITC and PI, and incubated in the dark on ice for 15 minutes before analysis. For cell cycle analysis, cells were fixed in ethanol at −20 °C overnight, treated with RNase A, resuspended in PBS, and incubated at room temperature for 15 minutes. Then, 100 µL of RNase A was added, and the cells were incubated at 37 °C for 30 minutes. Subsequently, 400 µL of LPI reagent (50 µg mL^−1^) was added, incubated in the dark for 30 minutes, and analyzed.

### Cell invasion assay

For the cell invasion assay, matrix gel was thawed from −20 °C to 4 °C and diluted 1 : 8 with serum-free medium. A volume of 65 µL per well was added to coat the chamber membrane, followed by incubation at 37 °C for 2 hours. When cells reached 80–90% confluence, they were digested with trypsin and adjusted to a concentration of 1 × 10^5^ cells per mL. A total of 700 µL of high-glucose DMEM with 20% serum was added to the lower chamber of the Transwell system, while 300 µL of cell suspension was added to the upper chamber. After 24 hours of incubation, the chamber was removed, and cells were fixed with 4% paraformaldehyde for 20–30 minutes before staining with 1% crystal violet for 30 minutes. Non-migrated cells in the upper chamber were removed using a cotton swab. Migrated cells on the membrane were observed under an inverted microscope.

### DNA platination

Cells were cultured in 6-well plates until they reached 90% confluence and then treated with oxaliplatin and BrPt3 for 12 hours. After washing with cold PBS and centrifugation, cells were resuspended in 1 mL of PBS. A 100 µL sample was taken to measure cell density. DNA was extracted using a Genomic DNA Mini Preparation Kit, and its concentration was determined. The extracted DNA was digested with 70% HNO_3_, and the platinum content was analyzed using ICP-MS. Platinum levels were expressed as nanograms of platinum per milligram of DNA.

### Glycolysis detection

Tumor cell glycolysis was monitored in real-time using the pH-Xtra™ Glycolysis Kit. Cells were seeded into 96-well plates at 5 × 10^5^ cells per well and cultured for 24 hours before drug treatment. According to the kit's protocol, the medium was removed, and cells were washed twice with 100 µL of respiration buffer. After the second wash, 90 µL of fresh buffer was added to each well, followed by 10 µL of pH-Xtra reagent, except for blank controls, which received 10 µL of buffer. Absorbance was measured at 590, 615, and 690 nm. Cells in the logarithmic growth phase were seeded into 6-well plates at 1.5 × 10^5^ cells per well and incubated for 24 hours. After 48 hours of drug treatment, cells were digested with trypsin, centrifuged at 1000 rpm for 5 minutes, and washed twice with PBS. The levels of lactate, pyruvate, ATP, and HK2 were measured using respective assay kits.

### *In vivo* antitumor effects

A total of 2 × 10^6^ HCT116 cells were subcutaneously injected into 5-week-old female nude mice. When tumors reached approximately 100 mm^3^, drug treatment was administered 5 days per week with a 2-day break for a total duration of 3 weeks. Tumor volume was measured every 3 days, and growth curves were plotted for tumor volume, relative tumor volume, and proliferation rate. Tissue homogenates were analyzed for lactate, pyruvate, glucose, ATP, and HK2 levels. Ki67 and γ-H2AX expression in tumor tissues was assessed using immunohistochemistry or immunofluorescence, while apoptosis in cancer cells was detected by TUNEL staining.

## Conclusions

The 3-bromopyruvic acid platinum(iv) complex (BrPt3) was designed and synthesized for cancer therapy. It demonstrates significant antitumor efficacy through dual mechanisms: inducing DNA damage and inhibiting glycolysis. These properties make it a promising drug candidate with oral bioavailability and the potential to overcome drug resistance.

## Ethical statement

All animal experiments were performed in accordance with the Guidelines for Care and Use of Laboratory Animals of Kunming Medical University and approved by the Animals Ethics Committee of Kunming Medical University (Approval No. kmmu20211185).

## Author contributions

Peng Zhou: conceptualization, methodology, investigation, writing – original draft. Qingfeng Xiong: conceptualization, methodology, investigation. Ling Zhang: investigation. Jing Jiang: investigation. Yan Song: investigation. Weiping Liu: investigation. Limei Zhang: investigation. Anli Gao: conceptualization, investigation, methodology. Chen Qing: conceptualization, writing – review & editing, funding acquisition, project administration, supervision.

## Conflicts of interest

There is no conflict of interest to declare.

## Supplementary Material

RA-015-D5RA02064F-s001

## Data Availability

The data supporting this article have been included as part of the ESI.†
