# Supplementary material for: Synthesis and anticancer activity of Pt(iv) prodrugs containing 3-bromopyruvic acid as an axial ligand
Source: RSC Adv. 2025 Jun 30;15(27):22063–75. doi: 10.1039/d5ra02064f (PMC12207602; doi:10.1039/d5ra02064f)
Supplement: RA-015-D5RA02064F-s001 [file RA-015-D5RA02064F-s001.pdf]

Supplementary Information (SI) for RSC Advances.
This journal is © The Royal Society of Chemistry 2025

Supporting Information

Synthesis and anticancer activity of Pt(IV) prodrugs containing 3-bromopyruvic acid as an axial ligand

Peng Zhou, ‡[ab] Qingfeng Xiong, ‡[c] Ling Zhang, [a] Jing Jiang, [c] Yan Song, [a] Weiping Liu,[c] Limei Zhang, [a] Anli Gao*[c] and Chen Qing*[a]

‡ These authors contributed equally to this work.

* Corresponding authors

Table of Contents **Page**

Table of Contents	Page
Chemical synthesis	2
Figure S1. ^{1}H NMR Spectrum of [Pt(DACH)(oxalate)(OH)$_2$]	3
Figure S2. ^{13}C NMR Spectrum of [Pt(DACH)(oxalate)(OH)$_2$]	3
Elemental analysis (calculated for BrPt3)	4
Figure S3. ^{1}H NMR Spectrum of BrPt3	4
Figure S4. ^{13}C NMR Spectrum of BrPt3	4
Figure S5. IR Spectrum of BrPt3	5
Figure S6. FAB^{+}-MS Spectrum of BrPt3	5
Single-cell gelelectrophoresis	6
Table S1 Impact of BrPt3 on tumor and organ coefficients in nude mice bearing HCT116 human colon cancer xenografts	7
Table S2 Effects of BrPt3 on the growth of transplanted HCT116 tumors and body weight in nude mice	8

Chemical synthesis

OXP (L20200428) was obtained from Kunming Guiyan Pharmaceutical Co. Ltd., while other chemicals were of analytical grade and used as received. All synthesis procedures were conducted under light-protected conditions. Elemental analyses for carbon, hydrogen, and nitrogen were performed using a Carlo-Erba instrument, while platinum content was determined following a previously reported method (EP 6.5). Fourier-transform infrared (FT-IR) spectra were recorded on a Nicolet iS10 spectrometer using KBr pellets, covering a range of 4000–400 cm^{-1}. Nuclear magnetic resonance (NMR) spectra ($^1H/^{13}C$) were obtained in DMSO-d_6 using a Bruker AVANCE III 500 MHz spectrometer with tetramethylsilane (TMS) as the standard. Mass spectra (FAB^+) were acquired using an AB Sciex Triple TOF mass spectrometers.

Synthesis of cis,trans,cis-Pt(lR,2R-diaminocyclohexane)(OH)(3-bromopyruvate) (C_2O_4)](BrPt3)

OXP (6 g) was dissolved in 600 mL of warm distilled water, followed by the slow addition of 21.5 mL of 30% H_2O_2 with continuous stirring for 5 hours. After a white precipitate formed, the mixture was coo led, filtered, washed with ice water, and dried at 60°C. The crude product was purified by recrystallizatio n in boiling water. The purified product was dissolved in 25 mL of water, and 5 mL of a 3-bromopyruv ic acid solution (0.86 g) was added. The mixture was stirred in a 35°C water bath for 48 hours until the solution cleared and a yellow solid formed. The solution was then concentrated under reduced pressure at 45°C until nearly dry. The product was filtered, washed twice with ice-cold acetone and ethanol, and vac uum-dried. The final yield of BrPt3 was 76.7% (2.07 g). Its structure is shown in Fig. 1A and was char acterized using elemental analysis, ^1H and ^{13}C nuclear magnetic resonance (NMR) spectroscopy, IR spectr oscopy and mass spectrometry (FAB^+-MS).

Anal. calcd. for $C_8H_{16}N_2O_6Pt$: Pt 45.24%、C 22.27%、H 3.71%、N 6.50%; found: Pt 45.18%、C 22.04%、H 3.91%、N 6.35%. ^1H NMR (500 MHz, DMSO-d_6) δ 7.72–7.67(m, 2H, NH_2), 6.91(s, 2H, NH_2), 3.32(s, H_2O), 2.49(d, J=3.1 Hz, DMSO), 1.98(d, J=12.3 Hz, 2H, 2CH-cyclohexyl), 1.46 (d, J=8.8 Hz, 2H, CH_2-c yclohexyl), 1.44–1.37(m, 2H, CH_2-cyclohexyl), 1.08 (d, J=10.6 Hz, 2H, $2CH_2$-cyclohexyl), 0.86–0.83(m, 2 H, $2CH_2$-cyclohexyl), -0.01 (s, TMS). ^{13}C NMR (126 MHz, DMSO-d_6) δ: 164.57(s), 60.40(s), 39.52(dp, J =42.1, 20.9 Hz), 30.51(s), 23.74(s);

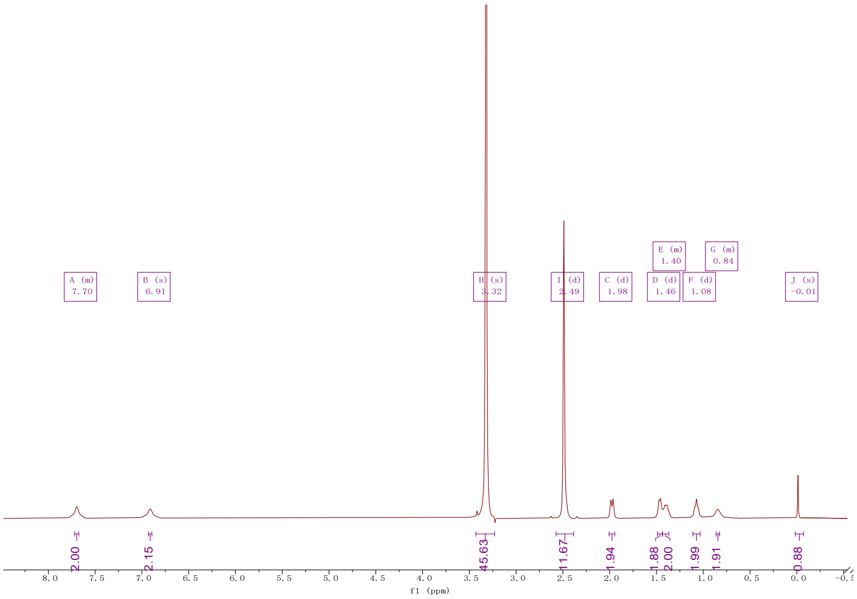

Figure S1. ^{1}H NMR Spectrum of [Pt(DACH)(oxalate)(OH)$_2$].

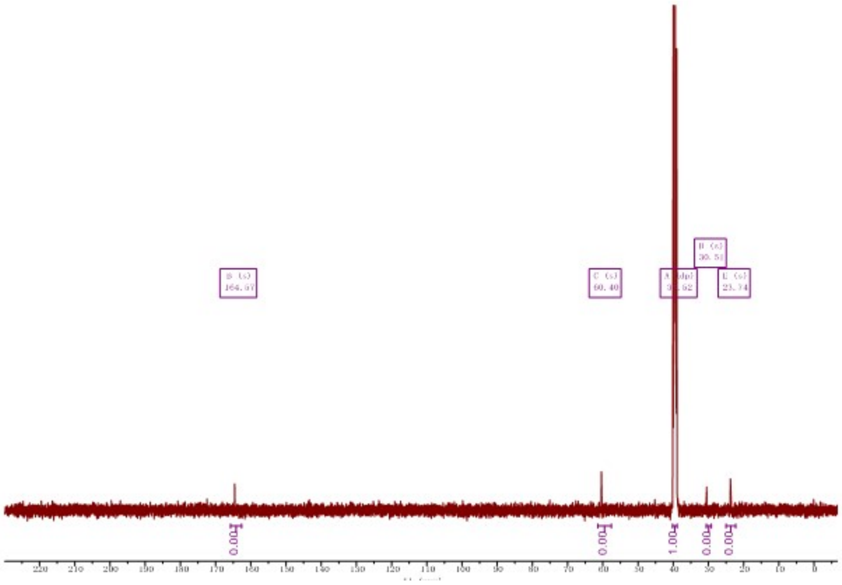

Figure S2. ^{13}C NMR Spectrum of [Pt(DACH)(oxalate)(OH)$_2$]

Elemental analysis (calculated for BrPt3): Pt 33.6%, C 22.8%, H 2.93%, N 4.82%; found: Pt 33.3%, C 22.6%, H 2.87%, N 4.78%.

^{1}H NMR (500 MHz, DMSO-d6) δ 6.19 (d, J=9.7 Hz, 2H，NH_2), 6.08 (d, J=9.9 Hz, 1H, NH_2), 5.74 (t, J=10.1 Hz, 1H, NH_2), 3.47–3.39 (m, 4H, H2O), 2.49(d, J=2.7 Hz, DMSO), 2.32 (q, J=11.7, 10.5 Hz, 2H, CH_2-3-BrPA), 2.00–1.84 (m, 2H, 2CH-cyclohexyl), 1.59–1.21 (m, 6H, 3CH_2-cyclohexyl), 1.19–0.94 (m, 2H, CH_2-cyclohexyl).

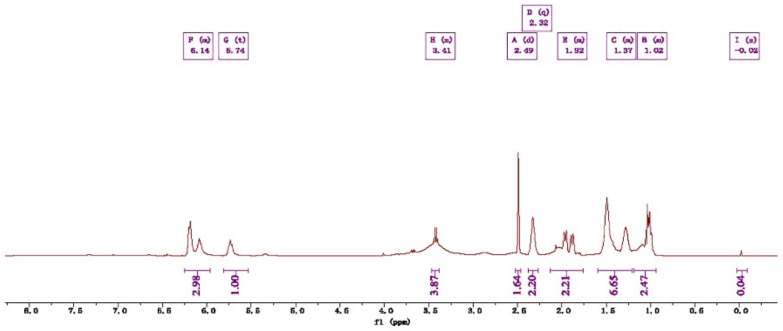

Figure S3. ^{1}H NMR Spectrum of BrPt3.

^{13}C NMR (126 MHz, DMSO-d_6) δ 165.95, 162.34, 100.44, 62.01, 61.12, 39.52 (dp, J=42.2, 21.1 Hz), 35.8 0, 31.54, 30.75, 24.08, 23.88, 0.12.

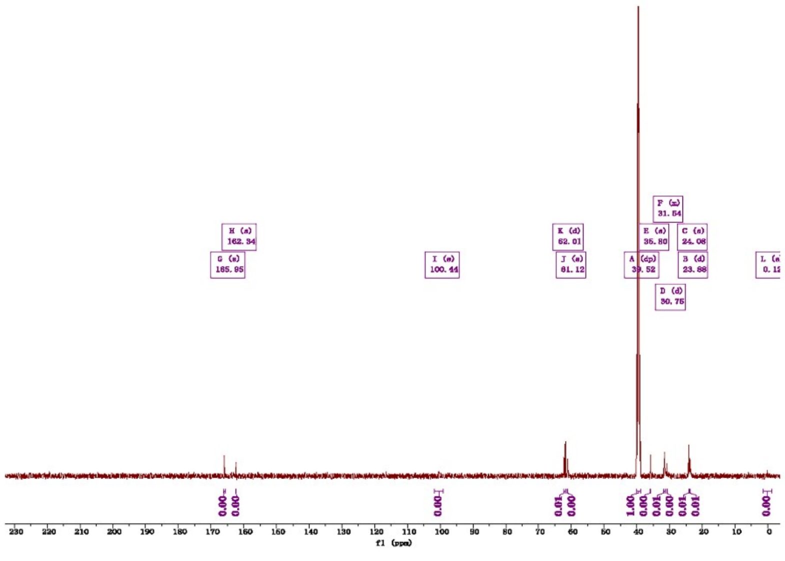

Figure S4. ^{13}C NMR Spectrum of BrPt3.

IR (cm⁻¹, KBr): 3547(s),3245(s), 3186(s),, 2933(s), 1718(s), 1624(s), 1562(m), 1449(s), 1397(s), 1378(s,), 601(w), 503(w)

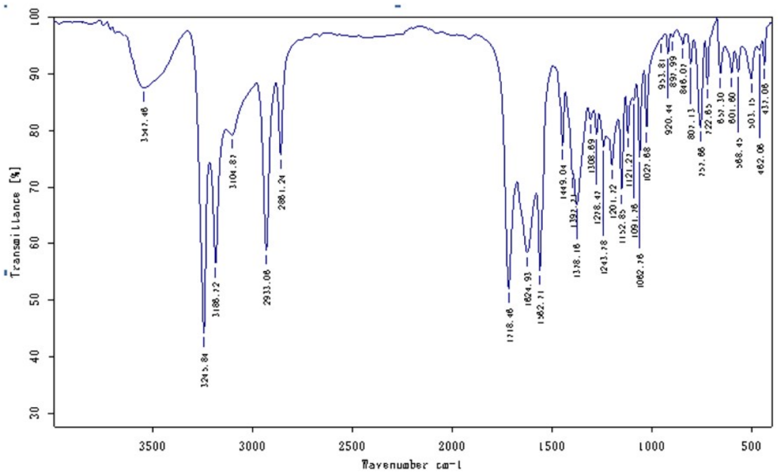

Figure S5. IR Spectrum of BrPt3.

FAB⁺-MS: m/z 580 $[MH]^+$.

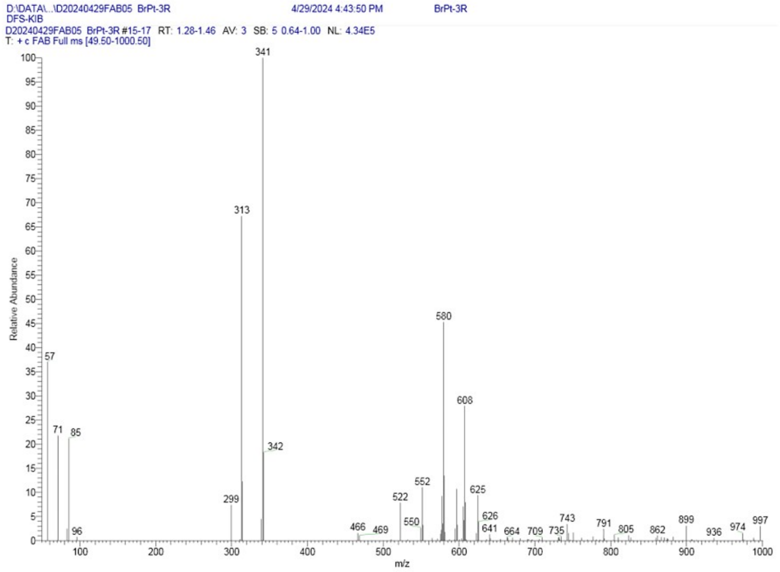

Figure S6. FAB⁺-MS Spectrum of BrPt3.

Single-cell gel electrophoresis

The cell suspension was centrifuged, and the supernatant was removed. The pellet was resuspended in PBS to a final density of 1×10^6 cells/mL. A 1% agarose gel layer was prepared on pre-warmed slides, covered with coverslips, and solidified at 4°C for 10 minutes. A mixture of 10 µL of cell suspension and 75 µL of 0.7% low-melting-point agarose (preheated to 37°C) was prepared and immediately spread as a second gel layer (70 µL) onto the first gel layer. The slide was solidified at 4°C for 10 minutes. A third layer of 75 µL preheated agarose (37°C) was added, covered with a fresh coverslip, and solidified at 4°C for 30 minutes. The lysis solution was prepared by mixing lysis buffer and DMSO at a 9:1 ratio. The slides were placed in a Petri dish, covered with 10 mL of lysis solution, and incubated overnight at 4°C. After lysis, the slides were rinsed with PBS for 3 minutes. Electrophoresis was performed by placing the slides in a tank filled with electrophoresis buffer for 60 minutes at room temperature, followed by electrophoresis at 25V for 20–30 minutes. The slides were then neutralized with buffer three times at 4°C for 5 minutes each. After discarding the buffer, 20 µL of propidium iodide solution was added, and the slides were stained in the dark for 10 minutes. The slides were then washed with ultrapure water, covered with coverslips, and prepared for fluorescence microscopy.

Table S1 Impact of BrPt3 on tumor and organ coefficients in nude mice bearing HCT116 human colon cancer xenografts(*n*=6)

Group	Dosage	Route of administration	Tumor weight（g）	Spleen index（mg/10g）	Liver coefficient（mg/g）	Kidney coefficient（mg/g）	Tumor inhibition rate（%）
control	0.1ml/10g	ip	2.31±0.38	52.05±7.68	69.54±2.66	15.73±1.42	—
OXP	7.6μmol/kg	ip	0.98±0.08****	26.00±3.21*	65.31±4.73	15.99±1.21	57.58
		ig	1.78±0.50	41.99±8.71	64.03±6.96	14.94±1.80	22.79
BrPt3	7.6μmol/kg	ip	0.68±0.39****	50.06±17.74	75.18±3.57	16.38±0.72	70.56
		ig	1.32±0.22**	45.78±14.22	68.32±6.31	15.84±1.51	42.78

Comparison with negative control group: *, $P<0.05$; **, $P<0.01$; ****, $P<0.0001$.

Table S2 Effects of BrPt3 on the growth of transplanted HCT116 tumors and body weight in nude mice(*n*=6)

Group	Dosage	Route of administration	Body weight（g）		TV（mm^3）		T/C（%）
			d_0	d_{21}	d_0	d_{21}	
control	0.1ml/10g	ip	20.67±1.01	20.13±0.64	36.51±4.46	1965.03±138.52	100.00
OXP	7.6μmol/kg	ip	21.83±0.53	18.75±0.80	49.70±11.84	898.34±542.75***	33.46
		ig	21.25±0.63	20.47±1.30	43.66±15.06	1950.48±343.42	82.70
BrPt3	7.6μmol/kg	ip	21.27±0.54	19.78±1.65	62.26±9.34	728.97±85.33****	21.67
		ig	21.08±0.54	20.68±0.92	51.39±11.79	1529.44±244.18	55.08

Comparison with negative control group: ***, P<0.001; ****, P<0.0001.
